# Elective laparoscopic splenectomy for giant hemangioma: a case report

**DOI:** 10.1186/1757-1626-2-10

**Published:** 2009-01-05

**Authors:** Christophoros Kosmidis, Christopher Efthimiadis, Georgios Anthimidis, Marios Grigoriou, Kalliopi Vasiliadou, Petros Sfikakis, Nikolaos Tziris, Epaminondas Fahantidis

**Affiliations:** 1 Department of Surgery, Interbalkan European Medical Center, Thessaloniki, Greece; 2 3rd Surgical Clinic, AHEPA Hospital, Thessaloniki, Greece; 3 1st Propeudeutic Surgical Clinic of A.U.Th., AHEPA Hospital, Thessaloniki, Greece

## Abstract

Although unusual, hemangioma is the most common primary splenic neoplasm. Splenectomy is indicated when the tumor is large, with increased risk of hemorrhage. The laparoscopic approach is preferred for most elective splenectomies. Although technically feasible, laparoscopic splenectomy can be a challenge in the patient with splenomegaly. We present herein a case of an 18-year-old male asymptomatic patient who underwent laparoscopic splenectomy for the incidental finding of splenomegaly caused by a large splenic hemangioma. Laparoscopic splenectomy appears to be a safe and effective procedure, in appropriately experienced hands, for patients with splenomegaly, given the spleen's fragile anatomy and its relationship to other abdominal viscera.

## Introduction

Splenic hemangioma is a rare disorder, but remains the most common benign neoplasm of the spleen [1]. Splenectomy is indicated because spontaneous rupture with massive hemorrhage can occur [2]. Laparoscopic splenectomy (LS) can be a challenging procedure given the fragile, well-vascularized nature of the spleen and its proximity to the pancreas, stomach and colon. Since the mid-1990s LS has steadily replaced open splenectomy as the approach of choice for most elective splenectomies [3]. We present herein a case of a patient who underwent LS for a hemangioma of the spleen.

## Case presentation

**Figure 1:**
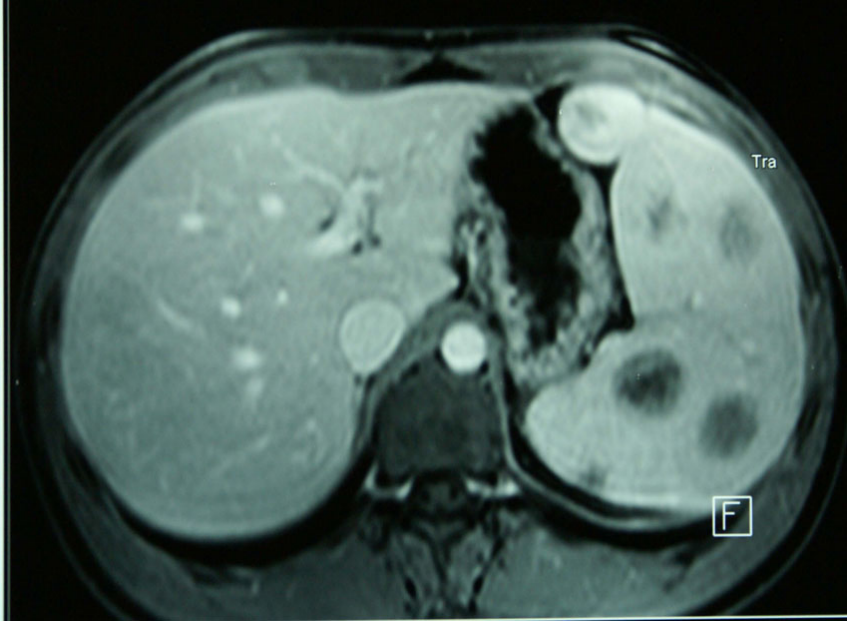
**Magnetic resonance imaging (MRI) showing the splenomegaly and the hemangioms**.

An 18-year-old Greek male student presented for screening test in order to get a health certificate. Physical examination revealed splenomegaly, although the patient was asymptomatic. No other clinical signs were detected. Ultrasonography and Magnetic resonance imaging (MRI) showed a hypoechogenic mass involving the spleen and the splenomegaly (Figure 1). Preoperative evaluation led to the possible diagnosis of hemangioma of the spleen. The patient was subjected to laparoscopy, which demonstrated a solid tumor of the spleen. Laparoscopic splenectomy was performed. Operative time was 65 minutes. Postoperative recovery was uneventful, the drain was removed on the second postoperative day and the patient was discharged on the same day. Histologic examination showed a capillary hemangioma of a spleen weighing 850 gr. The patient remains asymptomatic 6 months after the operation.

### Technique

Following induction of general anesthesia and endotracheal intubation, a nasogastric tube and a urinary catheter were inserted, and compression stockings were applied. The patient was placed on a beanbag in a 60-degree right lateral decubitus position. The right brachial plexus was protected with a pillow roll and the left arm was supported by a splint. The patient was positioned so that the table could be flexed to create a wider working space in a reverse Trendelenburg position. The surgeon and the scrub nurse stood to the patient's right, and the assistants stood to the left. The video monitors were placed on each side of the table, above the level of the patient's shoulders.

**Figure 2:**
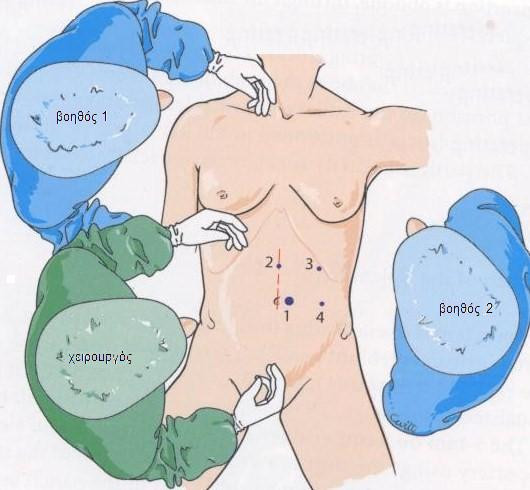
**Position of trocars**.

Pneumoperitoneum was established using a Veress needle to a pressure of 12 mm Hg. Four ports were used: the first 10-mm trocar was inserted at the left margin of the umbilicus. The other three 5-mm operating trocars were positioned as follows: the first trocar was on the median line, 8 cm above the umbilicus, a second operating trocar in a left subcostal position on the axillary line to enable gastroepiploic mobilization, and a left 5-mm operating trocar was positioned in the left lateral position, 10 cm from the umbilicus (Figure 2).

**Figure 3:**
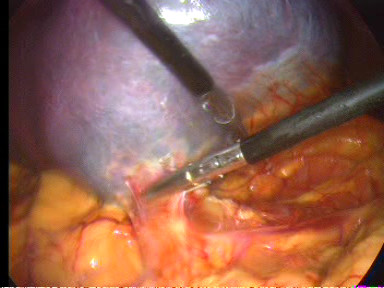
**Lift of the inferior pole of the spleen and division of the splenocolic ligament**.

**Figure 4:**
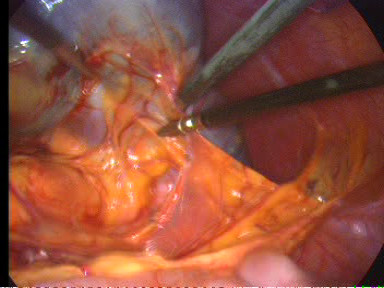
**Incision of the lateral peritoneal attachments of the spleen**.

**Figure 5:**
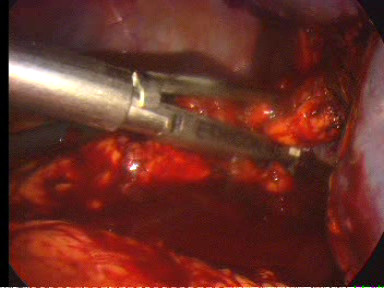
**Division of the vessels of the splenic hilum by application of endoscopic vascular staplers**.

The operation is begun with a thorough search of the abdominal cavity. The inferior pole of the spleen was lifted superiorly. The splenocolic ligament was divided (Figure 3). This mobilized the inferior aspect of the spleen and allowed the spleen to be retracted cephalad. Great care was taken to avoid rupture of the splenic capsule during retraction. The lateral peritoneal attachments of the spleen, the splenorenal and splenophrenic ligaments were sequentially incised (Figure 4). The splenic hilum was approached from the lower pole and dissection was continued cephalad. With the spleen elevated, the short gastric vessels and main vascular pedicle were visualized. The tail of the pancreas was also visualized and avoided at this point as it approached the splenic hilum. The splenic pedicle was carefully dissected from the medial and lateral aspects. After the short gastric vessels had been divided with ultrasonic dissector, the splenic artery and vein were dissected. The vessels were divided by application of endoscopic vascular staplers (ENDOPATH^®^, ETS Flex 45 Endoscopic Articulating Linear Cutter 45 mm staple line, 2.5 mm Staple Leg Length (Vascular/Thin). 45 MM Vascular/Thin). Each jaw was positioned anterior and posterior to the splenic vessels (Figure 5). The instrument was fired two times in sequence. To remove the detached spleen, a nylon extraction bag was introduced through the left lateral trocar site. The bag was opened within the abdominal cavity. An incision adequate to enable removal of the bag containing the intact spleen was made at the left lateral site. The spleen was placed into the specimen retrieval bag. The drawstring was grasped, and the bag was closed and drawn out. The laparoscope was reinserted and the splenic bed was assessed for hemostasis. Finally, peritoneal irrigation was carried out and a Rob drain tube N° 24 was placed at the residual cavity.

## Discussion

Most splenic hemangiomas are discovered incidentally, and their clinical importance generally lies in differentiating them from other conditions, particularly from metastases. Occasionally they may be associated with splenomegaly, abdominal pain, dyspnoea, diarrhea, or constipation. No potential for malignancy exists. Hemangiomas are not treated unless they are symptomatic or very large, with increased risk of hemorrhage; treatment is splenectomy [2]. In our case, the patient was asymptomatic and splenectomy was performed because of the large size of the spleen and the increased risk of rupture.

Many surgeons now prefer to use the laparoscopic approach for most elective splenectomies. In experienced hands, LS can be performed as safely and effectively as open splenectomy [4]. Operative time is longer for LS, but the procedure offers more rapid postoperative recovery and shorter duration of hospital stay [5]. Laparoscopic splenectomy can be completed in about 90% of properly selected patients. The incidence of conversion to open splenectomy is between 0% and 20% [5]. Most of the conversions are caused by intraoperative bleeding, but lack of surgical experience, extensive adhesions, large splenomegaly, and obesity are also involved [6-9]. A significant learning curve is observed with LS, and with increasing experience, the conversion rate has been reported to decrease dramatically [9-12]. The role of LS is now supported, in appropriately expert and experienced hands, for patients with splenomegaly, multiple prior abdominal surgeries, morbid obesity and the need for concomitant procedures [3]. Though technically feasible, LS in the patient with splenomegaly can be a challenge. Hand-assisted laparoscopic surgery has been suggested as a means by which splenectomy can be more safely and expeditiously performed in case of a large spleen [11].

Although the indications for LS are the same as for open splenectomy, some cases require caution. Absolute contraindications to the laparoscopic approach include severe cardiopulmonary disease and cirrhosis. Variceal short gastric vessels compounded by the coagulopathy of liver disease present an unacceptable risk for operative hemorrhage in patients with portal hypertension [5].

The earliest reports of LS detailed an anterior approach with the patient in the supine or in the low lithotomy position. Most procedures are now performed in the right lateral decubitus position. Some authors advocate a midway «double access» technique in which the patient is in a 45° right lateral decubitus position, facilitating the performance of concomitant surgery, such as laparoscopic cholecystectomy. Regardless of the position, the patient is placed so that the kidney rest can be raised to maximize the space between the iliac crest and the costal margin. As with the anterior approach, the double access technique requires the placement of five or six trocars. On the contrary, the lateral approach involves the use of three or four trocars and offers exposure of the anatomy in a way that allows for a more intuitive sequence of dissection, paralleling that of open splenectomy; the spleen is suspended from its diaphragmatic attachments, and gravity retracts the stomach, transverse colon, and greater omentum, while placing the splenic hilum under tension; after the short gastric vessels have been divided, the splenic pedicle may be carefully dissected from both the medial and lateral aspects; the surgeon easily visualizes the tail of the pancreas and avoids injury when placing the endovascular stapler [3,5].

The operation is begun with a thorough search of the abdominal cavity for the presence of accessory splenic tissue. Meticulous surgical technique to prevent splenosis secondary to splenic trauma during dissection is as important as a thorough search for accessory spleens [4]. A 1-cm cuff of peritoneum may be left along the lateral aspect of the spleen to act as a handle to manipulate the organ. The splenic vessels are divided following or prior to division of the short gastric vessels. The short gastric vessels can be divided by clips, endovascular stapling cartridges, ultrasonic dissection, diathermy or radio frequency ablation. For the control of splenic vessels ultrasonic dissector, hemoclips, bipolar devices, Liga-Sure, or an endovascular stapling device can be used. In our case a laparoscopic endovascular stapler, fired two times in sequence, was used (ENDOPATH^®^, ETS Flex 45 Endoscopic Articulating Linear Cutter 45 mm staple line, 2.5 mm Staple Leg Length (Vascular/Thin). 45 MM Vascular/Thin). The use of hemoclips is minimized throughout the procedure and especially around the hilum because the clips may interfere with future applications of a stapling device. The stapler will not function if a clip is caught within its jaws, and this can result in significant bleeding from hilar vessels. When possible, the splenic artery and vein are divided separately. However, mass hilar stapling is increasingly practiced with good long-term results. This is especially true for the magistral configuration of the splenic artery. The placement of a drain is not routinely recommended because it may be associated with postoperative left subphrenic abscess [3,5]. In our case a Rob drain tube was placed and subsequently removed on the second postoperative day. Our surgical technique has been described previously.

## Conclusion

LS is a safe procedure for patients with splenomegaly. Surgeons require advanced laparoscopic skills to deal with the challenge of splenectomy given the spleen's fragile anatomy and its relationship to other abdominal organs, especially the pancreas. Cautiousness is indicated in patients with portal hypertension and severe cardiopulmonary disease because they have a higher incidence of intraoperative bleeding and postoperative complications.

## Consent

Written informed consent was obtained from the patient for publication of this case report and accompanying images. A copy of the written consent is available for review by the Editor-in-Chief of his journal.

## Competing interests

The authors declare that they have no competing interests.

## Authors' contributions

CK, CE, MG, and GA analyzed and interpreted the data concerning the splenectomy operation. KV gathered the data concerning the patient case. PS, NT and EF contributed in the reference data. All authors read and approved the final manuscript.
